# Noise in the operating theatre

**DOI:** 10.1093/bjs/znab059

**Published:** 2021-02-28

**Authors:** T Hampton, S Everett, S Sharma, M Krishnan

**Affiliations:** 1 Ear, Nose and Throat Department, Alder Hey Children's NHS Foundation Trust, Liverpool, UK; 2 University of Liverpool, Liverpool, UK; 3 Acoustic Engineering, Hydrock, Manchester, UK

*Dear Editor*

Healthcare workers have reported difficulties in communicating within operating theatres as a result of using personal protective equipment (PPE) during the COVID-19 pandemic^1^. Here, the noise levels in the local operating theatre environment were assessed following best practice acoustic industry guidelines. Noise is defined by work safety regulations as a sound that may have a negative impact on health (such as impairment of hearing ability)^2^.

Noise is reported in the literature with various decibel (dB) weightings. Decibel is not a unit of loudness. It is log base 10 of the sound pressure (measured in pascals). Therefore, decibel is a relative logarithmic ratio to a reference sound level of 0 dB (where the sound pressure is 2 ×10^−5^ Pa or 0.000002 Pa), which is also the lowest sound pressure level that can be perceived by average human ears^3^. Where reported in healthcare or acoustic settings, dB is usually accompanied by an ‘A’ weighting (dBA) to indicate the sound frequency perception of humans.

Two meta-analyses^4^^,^^5^ have offered oversight on operating theatre noise. In 2010, Hasfeldt^4^ and co-workers reported on 16 papers assessing theatre noise, with average levels between 51 and 75 dBA, and maximum noise levels between 80 and 119 dBA. A meta-analysis by Wallis and colleagues^5^ in 2019 focused on measurement techniques rather than outcomes; among 76 studies measuring environmental noise in hospitals since 2008, they found that only 14.5 per cent achieved recommended technical reporting standards.

The present study is written in accordance with the Control of Noise at Work Regulations, which came into force for all industry sectors in Great Britain in 2005^2^. The noise level at which employers must provide hearing protection is 85 dB (daily or weekly L_Aeq_) and the level at which employers must assess workers’ health risk, and provide information and training, is 80 dB^2^.

Noise measurements were made using a class 1 integrating sound level meter (Fusion with Pre22 preamplifier and Cal31 calibrator 01dB; ACOEM, France; provided by Hydrock). The sound level meter was calibrated to 94 dB at 1 kHz both before and after the survey. Long-term noise measurements were made at the room perimeter, with a tripod and microphone placed approximately 1.2 m from the floor. Short-term noise measurements were made using class 2 multichannel noise dosimeters (40CD Microphone; G.R.A.S, Denmark; provided by Hydrock). The dosimeters were positioned at mid-chest height on surgeons, anaesthetists and circulating staff.

**Fig. 1:**
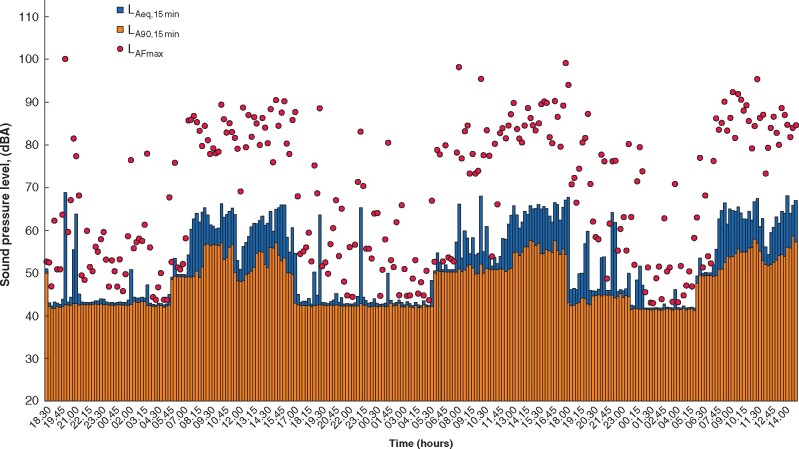
Noise recordings in operating theatre across 4 days Recordings were made from Tuesday 13 to Friday 16 October 2020. L_Aeq, 15 min_ (logarithmic average over 15 min); L_A90, 15 min_ (logarithmic average 90% of time over 15 min); L_AFmax_ (loudest peak throughout recording period).

Noise recording took place across 4 consecutive days during October 2020 (total 68 h). The L_AFmax_ (loudest) levels increased significantly with theatre use (*Fig. 1*). The in-use L_Amax_ was 100 dB, and the L_Aeq,12 h_ (logarithmic average over 12 h) was between 62 and 64 dB. The L_A90_ (average over 90 per cent of the time) values ranged from 53 to 54 dB, with a vacant theatre level of 41 dB. All values are comparable with those in existing literature, and Health and Safety Executive standards^2^. Staff dosimeters recorded a mean of +3 dB experienced by staff during procedures. Therefore, the L_Aeq_ level (or ambient noise) over which staff were communicating was approximately 65 dB.

Clarity is required, with reporting of terms such as peak, max, intensity, level, and average, which have explicit acoustic meanings that differ from common usage. The present findings echo suggestions to adopt standardized reporting guidelines for noise in operating theatres^5^. It is recommended that future studies report a minimum data set including L_A90_, L_Amax_, and L_Aeq_ in sound pressure levels (dBA weighting), not intensity. Documentation should report recording equipment manufacturer/model, location, and calibration. Clinician-mounted dosimeters should compare sound levels at the position of staff with central recording levels. An ambient noise L_Aeq_ of 65 dB should be considered for any communication devices or studies as the benchmark noise level over which healthcare staff communication can be assessed in operating theatres, particularly in an era of worsening communication owing to increasing use of PPE.
